# A Comparison of Smooth and Microtextured Breast Implants in Breast Augmentation: A Retrospective Study

**DOI:** 10.1055/s-0042-1760405

**Published:** 2023-03-28

**Authors:** Joo Hyuck Lee, Jae Hyuk Jang, Kyung Hee Min

**Affiliations:** 1 KIES-U Plastic Surgery Clinic, Seoul, Republic of Korea; 2 Department of Plastic and Reconstructive Surgery, Nowon Eulji Medical Center, School of Medicine, Eulji University, Seoul, Republic of Korea

**Keywords:** breast implants, mammaplasty, complications

## Abstract

**Background** The number of cosmetic and reconstructive surgeries that use breast implants is increasing in Korea. Recently, it has been reported that breast implant-associated anaplastic large-cell lymphoma is related to textured breast implants, and interest in classification according to the texture of breast implants is increasing. However, there is currently no clear and unified classification. In particular, the definition of “microtextured” is highly varied. In this study, we retrospectively investigated and analyzed the clinical outcomes of smooth and microtextured breast implants.

**Methods** A retrospective chart review of all patients who underwent breast augmentation surgery with smooth and microtextured silicone gel implants between January 2016 and July 2020 was performed. We retrospectively analyzed implant manufacturer, age, body mass index (BMI), smoking status, incision location, implant size, follow-up period, complications, and reoperation rate.

**Results** A total of 266 patients underwent breast augmentation surgery, of which 181 used smooth silicone gel implants and 85 used microtextured silicone gel implants. Age, BMI, smoking status, implant size, and follow-up period were not significantly different between the two groups. Similarly, complications and reoperation rates were not significantly different between the two groups.

**Conclusion** It is important to provide information regarding the clinical risks and benefits of breast implants to surgeons and patients through a clear and unified classification according to the texture of the breast implant.

## Introduction

**Fig. 1.**
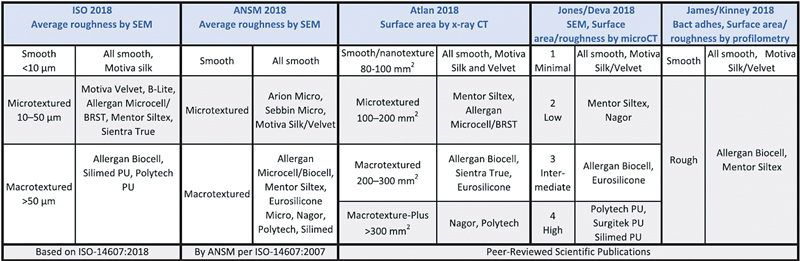
Summary of smooth and textured implant classifications. Surface area is a measure of the total area that the outer surface topography of an implant occupies and that interfaces with the patient. Surface roughness is a measure of the average height of the peaks and valleys of an implant surface. SEM, scanning electron microscopy; ISO, International Organization for Standardization; Bact adhes, bacterial adhesion. (Reprinted from Jones P, Mempin M, Hu H, et al. The functional influence of breast implant outer shell morphology on bacterial attachment and growth. Plast Reconstr Surg 2018;142:837–49.)

In Korea, cosmetic and reconstructive surgeries using breast implants are continuously increasing. 1 2 The clinical advantages and disadvantages of textured and smooth breast implants have been previously discussed. 3 4 5 6 7 8 9 10 Recently, it was reported that breast implant-associated anaplastic large cell lymphoma (BIA-ALCL) was associated with textured breast implants. 1 2 11 12 13 As interest in BIA-ALCL increases, various classifications for the textures of breast implants are being attempted and reported. 13 14 15 Textured breast implants are generally classified as macrotextured or microtextured. However, the real differences between them are complex and disordered. Many authors define the terms “macrotextured” and “microtextured” variably and arbitrarily. At present, no unified classification method has been developed. The industry-announced surface roughness values of Bellagel microtextured (HansBiomed Co., Ltd., Seoul, Korea) and Sebbin microtextured (Sebbin, Boissy-l'Aillerie, France) implants are 5.96 and 6 µm, respectively, and that of the Eurosilicone microtextured implant (Eurosilicone, Apt Cedex, France) is 24 µm. Sebbin and Bellagel microtextured implants follow the ANSM (Agence nationale de sécuritédu medicament) 2018 classification table, but they are both considered “smooth” if they follow the International Organization for Standardization (ISO) 2018 classification. In contrast, the Eurosilicone microtextured implant followed the ISO 2018 guidelines ( Fig. 1 ). 16 An absolute consensus has not yet been reached about the classification of “microtextured” implants. Therefore, we sorted the implants based on whether they had gross concavo-convex surfaces in this study. Mentor MemoryGel (Mentor Worldwide LLC, Irvine, CA) or Bellagel smooth implants were regarded as “smooth” because they do not have any gross concavo-convex surface, but Bellagel microtextured, Sebbin microtextured, and Eurosilicone microtextured implants were regarded as “microtextured” because they have observable concavo-convex surfaces.

Currently, the terms “microtextured” or “nanotextured” are used excessively without clear classification. In addition, these terms are used commercially without clinical basis, and inaccurate information is provided, which may cause confusion. Following identification of this problem, our study retrospectively investigated and analyzed the clinical results of smooth and microtextured breast implants. In addition, there have been many reports comparing smooth and textured breast implants, but few studies comparing smooth and microtextured breast implants.

## Methods

A retrospective chart review was performed for all patients who underwent breast augmentation surgery with smooth and microtextured silicone gel implants at a single plastic surgery clinic by a single surgeon between January 2016 and July 2020. Retrospective analysis was conducted, including implant manufacturer, age, body mass index (BMI), smoking status, incision location, implant size, length of follow-up, complications, and reoperation rate.

### Statistical Analysis

R language version 3.3.3 (R Foundation for Statistical Computing, Vienna, Austria) and T&F program version 3.0 (YooJin BioSoft, Korea) were used for all statistical analyses. For continuous variables, median (interquartile range) or mean ± standard deviation was computed, and the Mann–Whitney *U* test was performed to analyze differences between groups. Categorical variables are presented as sample numbers (%), and *p* -values were computed using Fisher's exact test or a two-sample proportion test to analyze differences between groups.

## Results

A total of 266 patients underwent breast augmentation surgery, of which 181 used smooth silicone gel implants and 85 used microtextured silicone gel implants. In the smooth silicone gel implant group, Mentor MemoryGel was used at 88.4%, and Bellagel was used at 11.6%. In the microtextured silicone gel implant group, Bellagel was used at 61.2%, Eurosilicone was used at 24.7%, and Sebbin was used at 14.1%.

Age, BMI, smoking status, implant size, and follow-up length were not significantly different between the two groups. Regarding the location of the incision, there were significantly more transaxillary incisions in the smooth implant group and significantly more transaxillary and inframammary incisions in the microtextured implant group ( *p* = 0.002) ( Table 1 ). There was no significant difference between the two groups in terms of the complication and reoperation rates. In the smooth implant group, complications occurred in 14.4%, and capsular contracture occurred in 1.7%. In the microtextured implant group, complications occurred in 8.2%, and capsular contracture occurred in 2.4%. Reoperation rate was 13.3% in the smooth implant group, and reoperation was performed in 8.2% in the microtextured implant group ( Table 2 ).

**Table 1:** Distribution of variables between the smooth implant group and micro-textured implant group

		Smooth	Microtextured	*p* -Value
Patients (%)		181 (68)	85 (32)	
Manufacturer				< 0.001 b
	Mentor	160 (88.4)	0 (0)	
	Bellagel	21 (11.6)	52 (61.2)	
	Sebbin	0 (0)	12 (14.1)	
	Eurosilicone	0 (0)	21 (24.7)	
Age (y)		33.94 ± 6.72	35.59 ± 7.72	0.178
BMI (kg/m ^2^ )		19.03 ± 1.65	19.37 ± 1.74	0.287
Smoking		63 (34.8)	24 (28.2)	0.328
Incision location				0.002 b
	Transaxillary	126 (69.6)	41 (48.2)	
	Inframammary	45 (24.9)	39 (45.9)	
	Periareolar	10 (5.5)	5 (5.9)	
Implant size (mL)		282.24 ± 32.94	285.32 ± 29.2	0.333
Follow-up length (mo)		11.35 ± 8.83	13.45 ± 10.63	0.356

Abbreviations: BMI, body mass index; SD, standard deviation.

Note: Values are presented as number (%) or mean ± SD.

a *p* -Value < 0.05.

b *p* -Value < 0.01.

**Table 2:** Comparison of complication and reoperation rates between the smooth implant group and microtextured implant group

		Smooth ( *n* = 181)	Microtextured ( *n* = 85)	*p* -Value
Complication		26 (14.4)	7 (8.2)	0.231
Complication type				
	Capsular contracture	3 (1.7)	2 (2.4)	1.000 a
	Bottoming out	12 (6.6)	1 (1.2)	0.106 a
	Malposition	1 (0.6)	0 (0)	1.000 a
	Asymmetry	1 (0.6)	1 (1.2)	1.000 a
	Hematoma	5 (2.8)	0 (0)	0.288 a
	Pain	2 (1.1)	0 (0)	0.832 a
	Rippling	1 (0.6)	0 (0)	1.000 a
	Double bubble	1 (0.6)	1 (1.2)	1.000 a
	Hypermobility	0 (0)	1 (1.2)	0.698 a
	Inflammation	0 (0)	1 (1.2)	0.698 a
Reoperation		24 (13.3)	7 (8.2)	0.306

Note: Values are presented as number (%).

a *p* -Values are computed using a two-samples proportion test to analyze the differences of each complication between the two groups.

In the microtextured implant group, according to the surface roughness value, Bellagel (5.96 µm) and Sebbin (6 µm) versus Eurosilicone (24 µm) were analyzed. Significant differences were found in terms of age, incision location, and follow-up length between the two groups. No significant differences were found concerning complications and reoperation rates ( Table 3 ).

**Table 3:** A comparison of variables between the Bellagel and Sebbin and the Eurosilicone groups in the microtextured implant group

		Bellagel and Sebbin	Eurosilicone	*p* -Value
Patients (%)		64 (75.3)	21 (24.7)	
Age (y)		34.20 ± 6.67	39.81 ± 9.23	0.012 a
BMI (kg/m ^2^ )		19.14 ± 1.43	20.07 ± 2.39	0.323
Smoking		21 (32.8)	3 (14.3)	0.161
Incision location				0.035 a
	Transaxillary	35 (54.7)	6 (28.6)	
	Inframammary	27 (42.2)	12 (57.1)	
	Periareolar	2 (3.1)	3 (14.3)	
Implant size (mL)		283.59 ± 29.45	290.60 ± 28.47	0.431
Follow-up length (mo)		15.41 ± 10.84	7.48 ± 7.37	0.003 b

Complication		5 (7.8)	2 (9.5)	1.000
Complication type				
	Capsular contracture	2 (3.1)	0 (0.0)	1.000 c
	Bottoming out	0 (0.0)	1 (4.8)	0.555 c
	Asymmetry	1 (1.6)	0 (0.0)	1.000 c
	Double bubble	1 (1.6)	0 (0.0)	1.000 c
	Hypermobility	0 (0.0)	1 (4.8)	0.555 c
	Inflammation	1 (1.6)	0 (0.0)	1.000 c
Reoperation		5 (7.8)	2 (9.5)	1.000

Abbreviations: BMI, body mass index; SD, standard deviation.

Note: Values are presented as number (%) or mean ± SD.

a *p* -Value < 0.05.

b *p* -Value < 0.01.

c *p* -Values are computed using a two-samples proportion test to analyze the differences of each complication between the two groups.

## Discussion

Since Cronin and Gerow first introduced silicone gel-filled implants in 1962, silicone gel implants have been developed into the fourth generation. These advancements have been directed toward preventing gel bleeding, implant rupture, and capsular contracture. 17 18 There is ongoing research and discussion about the clinical benefits and risks of implant surface texture (smooth vs. textured). Many studies have reported that textured implants have lower rates of capsular contracture, rippling, and malpositioning than smooth implants. 5 6 7 8 Capsular contracture causes pain and poor aesthetic results, requiring reoperation. The following three theories have been proposed regarding the effect of textured implants on the prevention of capsular contractures. The first is the degradation of the contracted capsule by cells on the surface of the textured implant; the second is the ingrowth of breast tissue into the texture of the implant, increasing friction and reducing synovial-type metaplasia; and the third is the disruption of the planar arrangement of fibroblasts and the vectors of contraction seen on the surface of smooth implants. 9 19 20 However, it has been reported that textured implants are somewhat related to the occurrence of a double capsule and late seroma. 9 10 BIA-ALCL, a rare type of T-cell lymphoma, is a unique iatrogenic disease with evidence of association with breast implants, especially textured implants. 1 2 11 12 13 With the recent increase in BIA-ALCL patients worldwide, the first BIA-ALCL patient was reported in Korea in 2019. 2 Therefore, interest in the texture of breast implants has increased, and studies have reported on a classification according to the textures of breast implants. 13 14 15 Currently, various classification methods are being used, as shown in Fig. 1 . 16 However, these classifications remain unclear. The same terms were used within various classification systems, without a uniform definition. In addition, manufacturers use these nonuniform classification systems for marketing. 3 16 Some manufacturers are commercially promoting that microtextured implants have the advantages of smooth and textured implants and compensate for the disadvantages. In our study, the clinical differences between smooth and microtextured silicone gel implants were compared, but there were no significant differences in complications or reoperation rates.

Moreover, in the microtextured implant group, no statistically significant differences in terms of complications or reoperation rates were observed between the two groups according to surface roughness values.

There are existing studies on smooth and microtextured silicone gel implants (as shown in the following paragraph), but our study holds clinical significance as there are few reports that completely compare the clinical results of smooth and microtextured silicone gel implants.

Buonomo et al conducted a study comparing the quality of life and aesthetic results of round smooth implants and shaped microtextured implants in breast reconstruction. They reported that round smooth implants had better softness and volume and less rippling. Additionally, shaped microtextured implants were found to be better in profile delineation. 21 Sforza et al evaluated the complication and reoperation rates of breast augmentation with two different Motiva silicone breast implants. A comparison of SilkSurface (nanotextured; mean surface roughness of 4,000 nm) and VelvetSurface (microtextured; mean surface roughness of 17 ± 3 µm) showed that the nanotextured SilkSurface had fewer complications than the microtextured VelvetSurface. 22 Han et al retrospectively studied the short-term safety of silicone gel breast implants used for breast augmentation in Korea. The study mainly compared various silicone gel implant products, but there was no comparison or analysis of smooth and microtextured silicone gel implants and no statistical significance was found between groups. 23 Tanner reported that the incidence of capsular contracture was low as a result of analyzing the clinical results of 214 patients who used microtextured silicone gel implants in breast cosmetic surgery. However, that study also did not compare smooth and microtextured silicone gel implants. 24

Based on our study findings, concern over whether to select a microtextured or smooth implant is not necessary. These two implants are marketed as being totally different; however, we found no clinical evidence to show that microtextured and smooth implants are manifestly different. ISO 2018 is cited frequently because many plastic surgeons are concerned about the association with the BIA-ALCL. While this classification has divided the microtextured and smooth implants separately, our findings did not support any such clinical difference.

Our study was limited, as it was conducted only in one clinic, the number of patients was not large, and only some implant products were compared. Based on the results of this study, we plan to conduct a comparative analysis by including more patients in the future. An international and uniform classification according to the textures of breast implants is required, and it is important to predict clinical benefits and risks according to the classification. Providing such reliable information to surgeons and patients may be helpful when discussing and determining surgery and breast implant options.

## References

[1] Collett D J Rakhorst H Lennox P Magnusson M Cooter R Deva A K Current risk estimate of breast implant-associated anaplastic large cell lymphoma in textured breast implants Plast Reconstr Surg 2019 143(3S A Review of Breast Implant-Associated Anaplastic Large Cell Lymphoma):30S 40S 30817554 10.1097/PRS.0000000000005567

[2] Kim I K Hong K Y Lee C K Analysis of the molecular signature of breast implant-associated anaplastic large cell lymphoma in an Asian patient Aesthet Surg J 2021 41 05 NP214 NP222 33367520 10.1093/asj/sjaa398 PMC8040250

[3] Wixtrom R N Garadi V Leopold J Canady J W Device-specific findings of imprinted-textured breast implants: characteristics, risks, and benefits Aesthet Surg J 2020 40 02 167 173 31121016 10.1093/asj/sjz155

[4] Maxwell G P Scheflan M Spear S Nava M B Hedén P Benefits and limitations of macrotextured breast implants and consensus recommendations for optimizing their effectiveness Aesthet Surg J 2014 34 06 876 881 25024450 10.1177/1090820X14538635

[5] Maxwell G P Van Natta B W Murphy D K Slicton A Bengtson B P Natrelle style 410 form-stable silicone breast implants: core study results at 6 years Aesthet Surg J 2012 32 06 709 717 22751081 10.1177/1090820X12452423

[6] Wong C H Samuel M Tan B K Song C Capsular contracture in subglandular breast augmentation with textured versus smooth breast implants: a systematic review Plast Reconstr Surg 2006 118 05 1224 1236 17016195 10.1097/01.prs.0000237013.50283.d2

[7] Stevens W G Nahabedian M Y Calobrace M B Risk factor analysis for capsular contracture: a 5-year Sientra study analysis using round, smooth, and textured implants for breast augmentation Plast Reconstr Surg 2013 132 05 1115 1123 24056498 10.1097/01.prs.0000435317.76381.68

[8] Jewell M L Jewell J L A comparison of outcomes involving highly cohesive, form-stable breast implants from two manufacturers in patients undergoing primary breast augmentation Aesthet Surg J 2010 30 01 51 65 20442075 10.1177/1090820X09360700

[9] Hall-Findlay E J Breast implant complication review: double capsules and late seromas Plast Reconstr Surg 2011 127 01 56 66 21200201 10.1097/PRS.0b013e3181fad34d

[10] Maxwell G P Brown M H Oefelein M G Kaplan H M Hedén P Clinical considerations regarding the risks and benefits of textured surface implants and double capsule Plast Reconstr Surg 2011 128 02 593 595 10.1097/PRS.0b013e31821eee8c 21788865

[11] Swerdlow S H Campo E Pileri S A The 2016 revision of the World Health Organization classification of lymphoid neoplasms Blood 2016 127 20 2375 2390 26980727 10.1182/blood-2016-01-643569 PMC4874220

[12] Brody G S Deapen D Taylor C R Anaplastic large cell lymphoma occurring in women with breast implants: analysis of 173 cases Plast Reconstr Surg 2015 135 03 695 705 25490535 10.1097/PRS.0000000000001033

[13] Magnusson M Beath K Cooter R The epidemiology of breast implant-associated anaplastic large cell lymphoma in Australia and New Zealand confirms the highest risk for grade 4 surface breast implants Plast Reconstr Surg 2019 143 05 1285 1292 30789476 10.1097/PRS.0000000000005500

[14] Barr S Hill E W Bayat A Functional biocompatibility testing of silicone breast implants and a novel classification system based on surface roughness J Mech Behav Biomed Mater 2017 75 75 81 28697402 10.1016/j.jmbbm.2017.06.030

[15] Valencia-Lazcano A A Alonso-Rasgado T Bayat A Characterisation of breast implant surfaces and correlation with fibroblast adhesion J Mech Behav Biomed Mater 2013 21 133 148 23545265 10.1016/j.jmbbm.2013.02.005

[16] Clemens M W Discussion: the epidemiology of breast implant-associated anaplastic large cell lymphoma in Australia and New Zealand confirms the highest risk for grade 4 surface breast implants Plast Reconstr Surg 2019 143 05 1295 1297 31033809 10.1097/PRS.0000000000005588

[17] Cronin T D Brauer R O Augmentation mammaplasty Surg Clin North Am 1971 51 02 441 452 5550701 10.1016/s0039-6109(16)39388-4

[18] Poeppl N Schreml S Lichtenegger F Lenich A Eisenmann-Klein M Prantl L Does the surface structure of implants have an impact on the formation of a capsular contracture? Aesthetic Plast Surg 2007 31 02 133 139 17205246 10.1007/s00266-006-0091-y

[19] Cash T F Duel L A Perkins L L Women's psychosocial outcomes of breast augmentation with silicone gel-filled implants: a 2-year prospective study Plast Reconstr Surg 2002 109 06 2112 2121, discussion 2122–2123 11994621 10.1097/00006534-200205000-00049

[20] Brohim R M Foresman P A Hildebrandt P K Rodeheaver G T Early tissue reaction to textured breast implant surfaces Ann Plast Surg 1992 28 04 354 362 1596069 10.1097/00000637-199204000-00010

[21] Buonomo O C Morando L Materazzo M Comparison of round smooth and shaped micro-textured implants in terms of quality of life and aesthetic outcomes in women undergoing breast reconstruction: a single-centre prospective study Updates Surg 2020 72 02 537 546 32062785 10.1007/s13304-020-00721-w

[22] Sforza M Zaccheddu R Alleruzzo A Preliminary 3-year evaluation of experience with SilkSurface and VelvetSurface Motiva silicone breast implants: a single-center experience with 5813 consecutive breast augmentation cases Aesthet Surg J 2018 38(suppl_2):S62 S73 29040364 10.1093/asj/sjx150 PMC5952962

[23] Han S Kim R Kim T S A preliminary retrospective study to assess the short-term safety of traditional smooth or microtextured silicone gel-filled breast implants in Korea Medicina (Kaunas) 2021 57 12 1370 34946315 10.3390/medicina57121370 PMC8705802

[24] Tanner B Low rate of capsular contracture in a series of 214 consecutive primary and revision breast augmentations using microtextured implants JPRAS Open 2017 15 66 73 32158801 10.1016/j.jpra.2017.10.007 PMC7061539

